# *Drosophila* Ten-m and Filamin Affect Motor Neuron Growth Cone Guidance

**DOI:** 10.1371/journal.pone.0022956

**Published:** 2011-08-08

**Authors:** Lihua Zheng, Yehudit Michelson, Vita Freger, Ziva Avraham, Koen J. T. Venken, Hugo J. Bellen, Monica J. Justice, Ron Wides

**Affiliations:** 1 Department of Molecular and Human Genetics, Baylor College of Medicine, Houston, Texas, United States of America; 2 Mina and Everard Goodman Faculty of Life Sciences, Bar-Ilan University, Ramat-Gan, Israel; 3 Program in Developmental Biology, Department of Neuroscience, and Howard Hughes Medical Institute (HHMI), Baylor College of Medicine, Houston, Texas, United States of America; MRC, University College of London, United Kingdom

## Abstract

The *Drosophila Ten-m* (also called *Tenascin-major*, or *odd Oz (odz))* gene has been associated with a pair-rule phenotype. We identified and characterized new alleles of *Drosophila Ten-m* to establish that this gene is not responsible for segmentation defects but rather causes defects in motor neuron axon routing. In *Ten-m* mutants the inter-segmental nerve (ISN) often crosses segment boundaries and fasciculates with the ISN in the adjacent segment. Ten-m is expressed in the central nervous system and epidermal stripes during the stages when the growth cones of the neurons that form the ISN navigate to their targets. Over-expression of Ten-m in epidermal cells also leads to ISN misrouting. We also found that Filamin, an actin binding protein, physically interacts with the Ten-m protein. Mutations in *cheerio*, which encodes Filamin, cause defects in motor neuron axon routing like those of *Ten-m*. During embryonic development, the expression of Filamin and Ten-m partially overlap in ectodermal cells. These results suggest that Ten-m and Filamin in epidermal cells might together influence growth cone progression.

## Introduction

During the development of vertebrate and invertebrate nervous systems, motor neurons project axons from the central nervous system (CNS) to reach their targets [1]. This complex, yet stereotyped, process is essential for numerous processes, including coordinated motor activity. The growth cones help motor axons to navigate and find their appropriate targets. As they migrate they sense and respond to guidance cues and make multiple path-finding decisions [2]. The guidance cues include attractive and repulsive factors [3], [4], which bind receptors on the growth cones, impact intracellular signaling pathways and intracellular states [5], and induce cytoskeletal rearrangements.

The fruitfly *Drosophila melanogaster* has been an excellent model to study motor axon guidance since the mechanisms underlying this process are highly conserved between invertebrates and vertebrates [6]. The *Drosophila* neuromuscular system is composed of ∼40 motor neurons and 30 muscles in each abdominal hemisegment. The 40 motor neurons have been individually characterized in terms of neuroblast lineage, axon trajectory and muscle target [7], [8], [9]. The motor neurons project axons from the CNS in two bundles, the intersegmental nerve (ISN) and the segmental nerve (SN), before defasciculating into five major branches [10]. Each branch innervates a subset of the 30 stereotypically-arranged muscles at distinct distances from the body wall and in specific dorso-ventral regions [7], [10]. Apart from the ISN and SN nerves, the smaller transverse nerve (TN) is a mixed motor and sensory projection with efferent axons that innervate at least two muscle fibers in the mid-body wall regions [11], [12].

Numerous genes have been shown to affect growth cone guidance. Genetic screens [10], [13] identified homozygous mutant embryos by using monoclonal antibodies against neural specific antigens and histological examination of their axonal projections [14], [15], [16]. Based on similar screens in *Drosophila* and *C. elegans*, as well as biochemical experiments, many key players in growth cone guidance have been identified. These include genes with roles only in axon guidance, and those encoding classic morphogens that also function as guidance molecules.

The Tenm/Odz gene family encodes transmembrane proteins that are highly conserved across all metazoan species. Two members (here called *Ten-m* and *Ten-a*) have been identified in *Drosophila* and four members, called Teneurins (Tenm-1 through Tenm-4) in chicken, mouse, rat and human. The proteins contain eight EGF-like domains and seven NHL repeats. The NHL repeats resemble WD repeats and may be involved in protein-protein interactions [17]. Although two mutually exclusive models for the topology of the Ten-m protein have been proposed [18], [19], one documented form of the *Drosophila* Ten-m is a type I, multiply cleaved heterodimeric transmembrane protein with extracellular EGF domains and a large C-terminal intracellular domain [20]. Comparison of the protein sequences in this family indicates that three domains show higher conservation than the remainder of the protein: the EGF-like domain, the NHL repeats, and the cysteine rich segment between the EGF and NHL repeat stretches.

*Drosophila Ten-m* was first identified as a pair-rule gene involved in segmentation during embryonic development [19], [21]. Its expression is dynamic during development, first as seven stripes in the cellular blastoderm, then as fourteen stripes from stage 12 onward [19], [21]. It is prominently expressed in the ventral nerve cord, the cardiac mesoderm and epidermis at late embryonic stages [19], [21] and it also expressed in all imaginal discs [22]. Functional information on the vertebrate homologues is accumulating. Chicken *Tenm-2* is expressed in the developing limb, somites and craniofacial mesenchyme [23]. Interestingly, its expression in a neuroblastoma cell line leads to excessive filopodia formation and enlarged growth cones [24]. Mouse *Tenm4/Odz4* is required for gastrulation, and is likely involved in a number of later developmental processes [25]. The *Tenm-4* homologue in rat (called Neurestin) is expressed in various types of neurons [26]. RNAi experiments in *C. elegans* have implicated Tenm-1 in axon guidance and cell migration [27]. The loss of *Tenm3* in mice affects guidance of retinal axons and generation of visual topography [28], and a number of allied processes [29].

To define the function of *Ten-m* in fruit flies we identified and characterized new mutant alleles and found no evidence for a pair-rule phenotype, in contrast to previous reports [19], [21]. Instead, both disruption, and epidermal over-expression, of *Ten-m* result in aberrant motor axonal path-finding. We also found that Filamin, an actin-binding protein, whose homologues are associated with various congenital malformations, in the brain cortex in humans, as well as in craniofacial, skeletal and visceral and urogenital tracts in vertebrates [30], interacts with Ten-m. Ten-m and Filamin partially co-localize in *Drosophila* embryos, and overlap in epidermal co-expression stripes. Disruption of the gene encoding Filamin, *cheerio*, results in similar aberrant motor axonal path-finding defects as those observed in *Ten-m* mutants. These results suggest that Ten-m and Filamin influence motor neuron migration either through their activites in the motor neurons, in the epidermis, or through both.

## Materials and Methods

### Fly strains, genetic and molecular methods

The following strains from the Bloomington stock center are used in this study: *y w*, *Canton S*, *P{PZ}Ten-m^05309^ ry^506^/TM3, ry Sb^1^*, here called “*5309*” [19], [21], [31], *y^1^w^67c23^; P{y^+mDint2^ w^BR.E.BR^ = SUPor-P}Ten-m^KG00101^ ry^506^/TM3, Sb^1^ Ser^1^*, here called “*N1*” [32]. *N1* is a SUPor-P (for suppressor-P element) gypsy-hybrid insertion [32], [33]. The insertion coordinate for *N1* was determined through *Hpa*II genomic DNA restriction, then inverse-PCR (http://flypush.imgen.bcm.tmc.edu/pscreen/). The PZ insertion of *5309* is at genome position 3L:22400915, and the P[SUPorP] insertion of *N1* is at genome position 3L: 22400924 (Flybase release 5.26). Imprecise excision and revertants of *N1* were generated by P-element excision according to established protocols [34]. Also used were: *Ten-m^CB04632^*, a GFP expressing insert driven by *Ten-m* control elements [35], *Ten-m^628^* [36], *Df(3L)Ten-m^AL1^/TM3, ry Sb^1^*, here called “AL1” [22], *Df(3L)Ten-m^AL29^/TM3, ry Sb^1^*, here called “AL29” [22], *w^1118^; Df(3L)Exel6138, P{w^+mC^ = XP-U}Exel6138/TM6B, Tb^1^*, here called “*Df (3L) Exel6138*” [37], *ru^1^ h^1^ th^1^ st^1^ opa^1^ cu^1^ sr^1^ e^s^ ca^1^/TM3, Sb^1^*, an *odd paired* allele, and *ru^1^ h^1^ th^1^ st^1^ opa^1^ cu^1^ sr^1^ e^s^ ca^1^/TM3, Sb^1^ Ser^1^* [38], [39], *cher^joy^* [40]; *cher^273^/TM6 Tb*, with an insertion from the Exelixis collection, and *cher^1^*/*TM3 Sb* [41]. *cherio* embryos were generated by crossing homozygous males with heterozygous *cher* females for alleles 273 and 1, or homozygous *cher^joy^* females, to yield 50% or 100% expected homozygotes, respectively. The following Gal4 drivers are used: *P{GawB}69B* [42]; and *paired*-Gal4. The 133 kb *Ten-m^+t133^* genomic fragment, gap-repaired in attB-P[acman]-Ap^R^, and integrated into the VK1 docking site is described in [43]. This fragment was additionally integrated into VK37 with a germline phiC31 source [44]. The frequency of rescue to viability was determined by scoring adults lacking balancer markers among total offspring derived from *Ten-m* balanced heterozygotes carrying the rescue element. The rescue of axon guidance defects (see below) in *Ten-m* mutant embryos was determined in offspring of *Ten-m* balanced heterozygotes carrying the rescue element, by assessing FasII staining in embryos lacking balancer markers, yet expressing transgene encoded Ten-m levels. The frequency of rescue reflected the extent of alleviation of the mutant phenotype in the homozygous *Ten-m* mutants carrying a rescue element.

Two directed deletions of virtually the entire *Ten-m* gene were generated utilizing FRT bearing P-elements inserted at the three prime end and five prime end of *Ten-m* [37]. The entire gene was deleted in *Ten-m^Δ525^*, which was generated through flippase mediated recombination between a third chromosome bearing PBac{WH}*Ten-m^f01130^*, and its homologous chromosome bearing P{XP}*Ten-m^d00619^*, by an established targeted deletion method [37]. *Ten-m^Δ510^*, which has all of *Ten-m* deleted except for the first exon, was generated in the same manner using a third chromosome bearing PBac{WH}*Ten-m^f01130^*, together with one bearing P{XP}*Ten-m^d06452^*. Lethal *Ten-m* alleles were either themselves marked with *lacZ* or GFP, or carried embryonic marked balancers or balancers that contribute no embryonic neuronal phenotypic effects. All *Ten-m* lethal heterozygous lines, alone or in inter-allelic combinations, give rise to an expected 25% *Ten-m* mutant embryos. For a comparison across all alleles, frequencies are presented uniformly in one table as the mutant phenotype rates within each entire offspring population. Lethal phase analysis was modified from that described [45] using a Kr-GFP balancer as in [46]. Cuticle preparation was slightly modified from the protocol described elsewhere [47].

### *Ten-m* transgenic fly generation

The Ten-m cDNA containing the whole open reading frame was cloned into the pUAST vector and transgenic lines were obtained according to established methods for P element transposition.

### Yeast two hybrid screening

The MATCHMAKER Two-hybrid System 3 (Clontech) was used for screening a *Drosophila* embryonic cDNA library (cloned into the pACTII vector, Steve Elledge, Harvard University). The bait was the cytoplasmic region of the *Drosophila Ten-m* gene (nucleotides 3423 to 8891 in X73154), which was cloned into the pGBKT7 vector (Clontech). Positive colonies were identified as those being able to grow on the quadruple dropout media (-trp/-leu/-his/-ade). The positive interactions were then confirmed in beta-galactosidase overlay assays. The candidate plasmids were rescued by electroporation into *E. coli* cells and then sequenced.

### Deletion mapping in yeast

The cytoplasmic region of Ten-m was truncated into several segments and cloned into the pGBKT7 vector. Based on X73154, these are the truncated segments: Seg1, Nt. (nucleotide) 3423–4213; Seg2, Nt. 4213–5193; Seg3, Nt. 5193–8891; Seg4, Nt. 3423–5193; Seg5, Nt. 4213–8891 and Seg6, Nt. 3423–8891. Deletion mapping was carried out by co-transforming two plasmids (Ten-m fragment in pGBKT7, Filamin fragment in pACTII) into the AH109 yeast strain (Clontech). Equal amounts of transformed cells were plated on selective media. The ratio (as a percentage) of the number of colonies that grow on quadruple dropout media (-trp/-leu/-his/-ade + 2.5 mM 3-amino-1,2,4-triazole) over the number of colonies that grow on double dropout media (-trp/-leu) was calculated.

### GST pull down assay

GST fusion protein constructs were made with the pGEX4T2 vector (Stratagene). The GST-Filamin fusion proteins, or GST alone, were expressed in BL21 (Stratagene) upon induction by isopropyl thiogalactoside (IPTG) and purified by incubating the resulting bacterial cell lysate with equilibrated Glutathione-sepharose beads (Pharmacia). The beads were washed five times with binding buffer (0.5% NP40 in PBS supplemented with EDTA free protease inhibitor cocktail from Roche). Truncated Ten-m segments 1, 2, and 4 were *in vitro* translated and ^35^S labeled with TNT T7 coupled reticulocyte lysate system (Promega). The fusion protein-bound beads and the ^35^S labeled proteins were mixed, rotated for 2 hours at 4 degrees, washed five times with the binding buffer, then resolved on an SDS PAGE gel that was subsequently fixed, dried and exposed to X-ray film.

### Whole mount immunohistochemistry

*Drosophila* embryos were collected, aged, fixed in 3.7% or 4% formaldehyde and blocked in 5% normal goat serum in PBT (0.1% Tween20 in PBS) according to standard procedures. The following primary antibodies were used in this study: monoclonal anti-Ten-m antibody (Mab113) 1∶300 [21]; monoclonal anti-Ten-m antibody (Mab20) 1∶3000 [19], rat anti-Filamin polyclonal antibody 1∶3000 [48](Lynn Cooley, Yale University); anti-fasciclin II (1D4) 1∶5 [13](obtained from the Developmental Studies Hybridoma Bank (DSHB)); BP102 1∶20, [13](DSHB), 22C10 1∶20 [15](DSHB), and rabbit anti-beta galactosidase 1∶5000 (MP Biomedicals). The embryos were washed in PBST four times for 15 minutes each, then stained with secondary antibodies: biotinylated anti-mouse antibody 1∶400 and biotinylated anti-rat antibody 1∶400 (Vector Laboratory); Alexa488-anti-mouse 1∶400 (Molecular Probes); and Cy3-anti-rat 1∶500 (Jackson ImmunoResearch Laboratories). The embryos were washed again for four times, 15 minutes each and treated in the following way: for the biotinylated staining, a and b solutions (Vector Laboratory) were added and color reactions were performed according to the manufacture's protocol; for fluorescent staining, as above or the embryos were filleted and mounted in 70% glycerol.

## Results

### New *Ten-m* mutations, and phenotypic rescues with a genomic construct, show that *Ten-m* is not a segmentation gene

**Figure 1:**
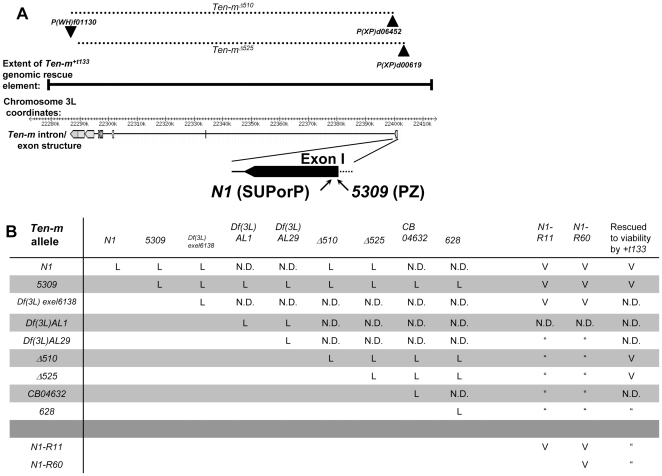
*Ten-m* N1, new deletions, and known Ten-m alleles are allelic, lethal, and P[acman] construct rescued. (**A**) A schematic displaying the introns and exons of *Ten-m* appears just below the coordinates of the *D. melanogaster* 3L chromosome arm, (Release 5.26, in kilobases), below which the positions of the P-element insertions of *Ten-m* alleles *5309* and *N1* appear in an expanded view of exon 1. Above the 3L coordinates is a representation of the extent of the genomic rescue element contained in *Ten-m^+t133^*. Above that, the positions of the three FRT bearing P-elements used to produce *Ten-m* deletions *Ten-m^Δ510^* and *Ten-m^Δ525^*, and the extent of those deletions as dashed lines, are represented. (**B**) Complementation tests for viability were conducted among available alleles, deletions, and P-element excisions. L, lethal; V, viable or rescued by Ten-m transgene, N.D., not determined.

We found that a homozygous lethal P-element insertion, named *P^KG0010^*, abbreviated *N1*, was inserted in the *Ten-m* locus [32]. The P-element is inserted in the first exon of *Ten-m* [19], [21], one of the largest fly genes (Figure 1A). This insertion site is 34 nucleotides downstream of the transcription start site in the 5′ UTR, very close to the P-element insertion site of the most studied *Ten-m* allele, *Ten-m^l(3)05309^*, abbreviated Ten-m*^5309^*, or *5309* (Figure 1A). *N1* failed to complement the lethality associated with *5309* (Figure 1B), and as had been shown for *5309*, is a Ten-m protein null (Supporting Information S1). To demonstrate that the lethality is associated with the *N1* insertion, we excised the P-element and reverted the lethality (lines *N1-R11* and *N1-R60*). We also generated deletions of *Ten-m* (*Ten-m^Δ510^* and *Ten-m^Δ525^*, see Figure 1A) using the flippase-mediated directed deletion approach [37], with P-element pairs shown in Figure 1A. The *N1* line also fails to complement the lethality of these two *Ten-m* deletions, or a larger previously generated deletion covering the region, *Df (3L) Exel6138* (Figure 1B). The revertants *N1-R11* and *N1-R60* complement *Df (3L) Exel6138*, further supporting that the lethality of *N1* is caused by the insertion (Figure 1B).

The homozygous lethality associated with the *Ten-m^5309^* and *Ten-m^N1^* P-element insertion mutations, as well as the *Ten-m^Δ510^* and *Ten-m^Δ525^* deletions, was rescued using a P[acman] vector containing a 133 kb genomic rescue construct, *Ten-m^+t133^* (Figure 1A, B) [43]. These data demonstrate that *N1* is a *Ten-m* allele, that the lethality associated with the above *Ten-m* alleles is solely due to mutations in *Ten-m*, and that these alleles correspond to severe loss of function or null alleles.

**Figure 2:**
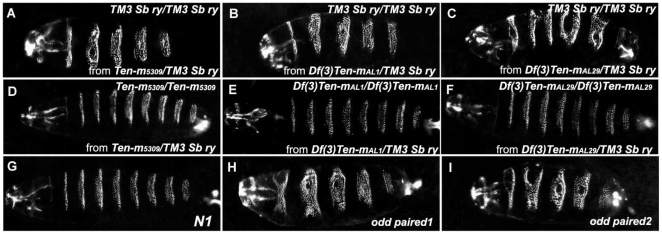
Tenascin-m is not a pair-rule gene. (**A–I**) Cuticles of dechorionated and devitellinized embryos were prepared to visualize exoskeletons and segmentation phenotypes. The cuticle phenotypes in the original *Ten-m* mutant stock and *odd paired* alleles are shown. The new *Ten-m N1* allele and rebalanced reference alleles fail to reproduce a pair-rule phenotype. Embryos shown are derived from the original: *5309* (A and D) ; *Df(3L)Ten-m^AL1^* (B and E); and *Df(3L)Ten-m^AL29^* (C and F) stocks. (**G**) *N1*, (**H**) *odd paired^1^*, and (**I**) *odd paired^2^*. Embryos are oriented: anterior-left.

Interestingly, no patterning defects were observed in *N1* embryos (Figure 2G), contrary to previous data indicating that *Ten-m* is a pair-rule gene [19], [21]. We rebalanced the *Ten-m^N1^* mutation with a GFP marked balancer chromosome (*Kr-GFP*) [46], and examined the lethal phase. A majority of homozygous *Ten-m^N1^* mutants hatched and survived to the first larval stage, and some survived to the second larval stage. *Ten-m^5309^* showed the same lethal phase when a different balancer was used than the one in the original stock. These data are not in agreement with the lethal phase of *Ten-m* mutations, previously reported to be embryonic lethal [21]. We therefore prepared cuticle preparations of embryos of the *5309* allele in the original balanced stock (*Ten-m5309/TM3, Sb ry*), as well as two X-ray induced deletion alleles, named *AL1* and *AL29*, which are propagated with the same balancer (e.g. *Df(3L)Ten-m^AL1^/TM3, Sb ry*) [22]. All three stocks produced embryos with obvious patterning defects as reported previously [22]. To determine if the segmentation defects were associated with the *Ten-m* mutations or with mutations on the balancer chromosome, we rebalanced the *5309*, *AL1* and *AL29* mutations with another balancer (*TM3, Sb*). Surprisingly, none of the rebalanced stocks produced embryos with cuticle phenotypes (Figure 2D, E and F). Moreover, the balancers from the original three stocks produced severe cuticle defects when outcrossed and homozygosed (Figure 2A, B and C). These results demonstrate that the cuticle fusion phenotype in the original *5309*, *AL1* and *AL29* stocks are caused by a mutation present on this *TM3, Sb ry* balancer, and that mutations in *Ten-m* do not lead to a pair-rule like defect.

Since the cuticle defects in these balancers are very similar to those associated with *odd-paired* (*opa*), a third chromosome pair-rule gene [39] (Figure 2H and I), we carried out complementation tests between the *TM3, Sb ry* balancer and *odd-paired* mutations. The balancer chromosome fails to complement *odd-paired* mutations (data not shown). We conclude that the *odd*-*paired* gene on the balancer chromosomes is mutated, and that it caused the segmentation defects observed in the original *5309*, *AL1* and *AL29* lines.

### *Drosophila Ten-m* mutants show peripheral motor neuron axon guidance defects

As Ten-m is prominently expressed in the nervous system, we stained mutant embryos with neuronally directed antibodies (BP102, 22C10, 1D4), and discovered specific axon guidance defects. The commissural axons (visualized with BP102) do not exhibit defects in *Ten-m* mutants (data not shown). Sensory neuron placement, their overall morphology, and their axons also display no significant aberrations (visualized with 22C10, data not shown). However, staining with Mab 1D4, which recognizes the neural cell adhesion molecule Fasciclin II and labels motor axons [10], revealed severe motor axon routing defects in *Ten-m* mutants.

**Figure 3:**
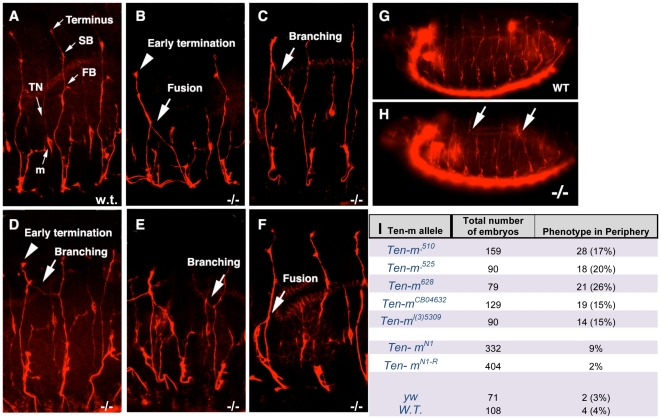
Motor neuron axon guidance defects in mutant *Ten-m* embryos. The mutant embryos show severe defects in motor neuron axon navigation as shown by motor axon specific marker anti-fasII staining. (**A–G**) The embryos examined are all at stage 16, anterior - left, dorsal - up. TN: transverse nerve. m: a characteristic cell lying along transverse nerve. The arrows refer to the defasciculation of ISN and the arrowheads to the premature termination of ISN. FB, first branch; SB, second branch. (**A**) Wild type. ISN axons have been fully developed with three characteristic branches (FB, SB and Terminal growth cone). (**B–F**) *Ten-m* mutants. The adjacent ISNs are fused at different positions along the axon with excessive branching. The fusion point could be below FB (**B**) or around SB (**C**). The adjacent ISNs could be connected with a bridge (**D**). Some ISNs have the “Y” shape (**E**). In other cases, the transverse nerve travels along the ISN (**F**). (**G**) Overview of wild type whole embryo. (**H**) Overview of mutant whole embryo. Occasionally there are two fusions in one embryo. (**I**) Penetrance of the mutant phenotype. The percentage (in parentheses) is the number of mutant embryos that show axon fusion out of the total number of embryo offspring derived from balanced stocks. For the lethal *Ten-m* lines, approximately 25% of offspring are expected to be *Ten-m* homozygotes. For the viable *Ten-m^N1-R^*, wild type, and *y w* lines, all offspring are of the same genotype.

The *Drosophila* peripheral motor axon projections have a stereotypical pattern [49]. The ISN navigates away from the CNS at embryonic stage 14 and reaches its terminus (near muscle 1) at stage 16 [10]. The ISN has three characteristic side branches: the first branch (FB) at the junction of muscles 3 and 11, the second branch (SB) at the junction of muscles 2 and 10 and a terminal branch at the junction of muscles 1 and 9 (Figure 3A, G) [10], [49]. In *Ten-m* mutant embryos, the ISN projections exit the CNS normally but aberrant patterns are observed at different extension points when they reach the dorsal or dorsal-lateral region of the body wall (Figure 3B–F, H). These aberrant patterns can be subdivided into at least four different groups. First, some ISNs cross the segmental boundary and fasciculate with adjacent ISNs (Figure 3B). Second, there are ISNs that defasciculate below the FB and the terminus. In some cases, the defasciculated branch crosses the segmental boundary and fasciculates with an adjacent ISN (Figure 3C and D). In others, the defasciculated branch crosses the segmental boundary and remains defasciculated (Figure 3E). Third, some ISNs fail to reach the terminal domain in the dorsal muscle domain (Figure 3B and D). Fourth, the transverse nerve will occasionally fasciculate with an ISN (Figure 3F).

These defects do not occur in all embryos nor do they occur in all segments. As an example, 68% of *Ten-m^Δ510^* homozygotes, distinguishable from their siblings by their lack of *lacZ* marked balancers, display defects in axons of at least one segment. Almost half of these have defects in three or more segments, (cases with two and four segments are shown in Figure 3G and H). The defects documented in a series of *Ten-m* alleles are shown in Figure 3I. The different alleles display phenotypes in 8.5 to 25% of all offspring of lethal *Ten-m* balanced lines (Figure 3I) (where 25% among all of the offspring are expected to be *Ten-m* homozygotes). Viable P-element revertants (*Ten-m^N1-R^*) do not exhibit motor axon defects (Figure 3I) (where 100% of the offspring are *Ten-m* homozygotes), demonstrating that the *Ten-m* lesions are responsible for the observed motor axon phenotypes. *Ten-m* mutant embryos for two transallelic combinations (*Ten-m^510^/Ten-m^CB04632^* and *Ten-m^l(3)5309^/Ten-m^CB04632^*) displayed the axon guidance defects at a 60% frequency, close to the frequency of each homozygosed allele. The axon guidance defects are also rescued by the P[acman] genomic *Ten-m^+t133^* transgene (not shown). Approximately 25% of Ten-m homozygotes are rescued to adult viability by a single transgene element, and the extent of alleviation of the FasII scored axon defect phenotypes is similar. We did not observe defects in the *Ten-m* embryos of the various alleles when they were stained with Mab 22C10 and BP102, suggesting that the motor neurons are specifically affected. In addition, we did not observe segmentation, tracheal, or muscle defects (data not shown).

### Ectopic expression of Ten-m results in motor neuron axon guidance defects

**Figure 4:**
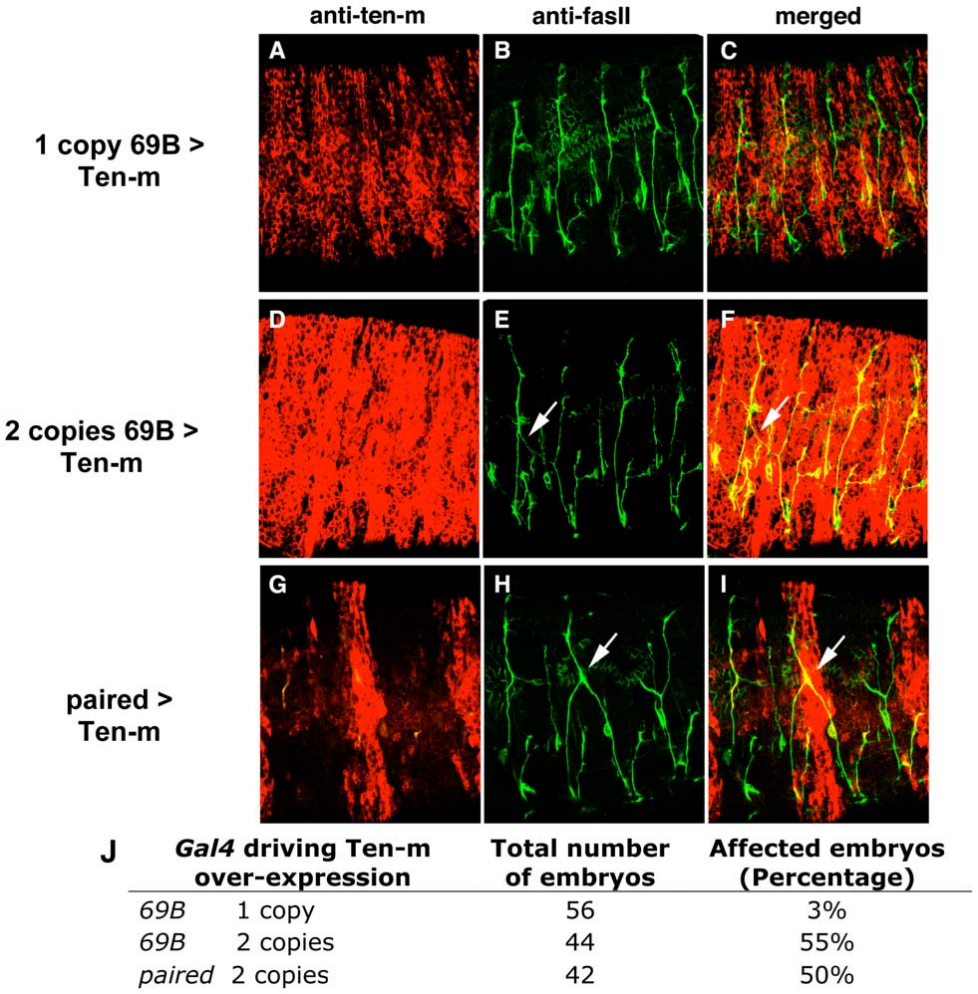
Motor neuron axon guidance defects in embryos over-expressing Ten-m. Stage 16 embryos are stained with Ten-m and FasII antibodies, anterior - left, dorsal -up. Arrows refer to the ISN segmental crossings. (**A–C**) Control embryos with low-level Ten-m expression driven by one copy of 69B GAL4. The ISN axon routing is normal. (**D–F**) Embryos with high-level Ten-m overexpression driven by two copies of 69B GAL4. The ISN de-fasciculates in one segment, crosses the segmental boundary and fasciculates with the ISN in the nearby segment. (**G–I**) Embryos with high-level Ten-m expression in every other segment driven by paired GAL4. Excessive defasciculation occurs in these embryos. ISN axons cross segmental boundaries and fasciculate together. Embryos are stained with Ten-m and FasII antibodies. Paired-Gal4/UAS-Ten-m embryos also display a high frequency of defects. (**J**) The percentage is the number of stage 16 embryos that exhibit axonal defects versus the total number of embryos that over-express Ten-m.

The crossover or early termination phenotypes of ISN occur laterally and dorsally, with initial motor axon outgrowth from the CNS unaffected. This is reminiscent of phenotypes for mutations of genes providing distal cues for motor axons with migration defects [1], [6], [50], [51]. These include genes whose expanded peripheral or pan-muscle over-expression produce phenotypes similar to their loss of function phenotypes, suggesting they encode instructive cue molecules with localization essential for proper motor axon migration [50]. Beyond its abundant CNS presence, Ten-m is also expressed in stripes from stage 12 [19], [21], as shown in embryos at late stage 14 (below, Figure 4A and 7B, Supporting Information S1). To determine if ectopic Ten-m expression impacts ISN axon migration, we used the GAL4/UAS system [42] to ectopically express Ten-m throughout the epidermis starting from embryonic stage 9 (see Materials and Methods). Driving expression of Ten-m throughout the epidermis using a single copy of the ubiquitous 69B driver [42], causes no obvious phenotype (Figure 4A–C, J). However, ∼55% of embryos that carry two copies of the 69B GAL4 driver exhibit ISN migration defects, including abnormal defasciculation, segmental boundary crossovers, as well as bifurcations at the termini (Figure 4D–F, J). To test whether Ten-m overexpression in epidermal stripes can cause these defects, we overexpressed Ten-m in every other segment, using the paired-GAL4 driver. In ∼50% of these embryos, we observed abnormal axon guidance defects (Figure 4J). As shown in Figure 4G–I, ectodermal stripes in which Ten-m is overexpressed exhibit a defect in the ISN, often crossing segmental boundaries and fasciculating with the ISN in the adjacent segment. In some cases, the ISN does not cross segmental boundaries but defasciculates and migrates abnormally in the same segment (Figure 4G–I). Hence, ectopic expression of Ten-m in ectodermal cells perturbs axonal guidance, suggesting a possible role for ectodermal Ten-m in axon-guidance.

### *Drosophila* Ten-m interacts with Filamin

Ten-m has been proposed to have several topologies [18], [20]. One of these topologies places the amino-terminal end of Ten-m extracellularly and the carboxy-terminal end in the cytoplasm (Type I topology). To identify intracellular proteins which bind to Ten-m, we carried out a yeast two-hybrid screen using the presumptive intracellular part of Ten-m (amino acid 910–1822) as bait and identified Filamin as an interaction partner from a *Drosophila* embryonic cDNA library (Supporting Information S1). We obtained seven positive colonies corresponding to five different clones for Filamin. Among the five clones, the 180 amino acids at the C-terminus of the Filamin protein were sufficient for the interaction. The interaction between Ten-m and Filamin was also represented in a *Drosophila* whole proteome matrix ([52]; http://portal.curagen.com/cgi-bin/interaction/flyHome.pl.).

**Figure 5:**
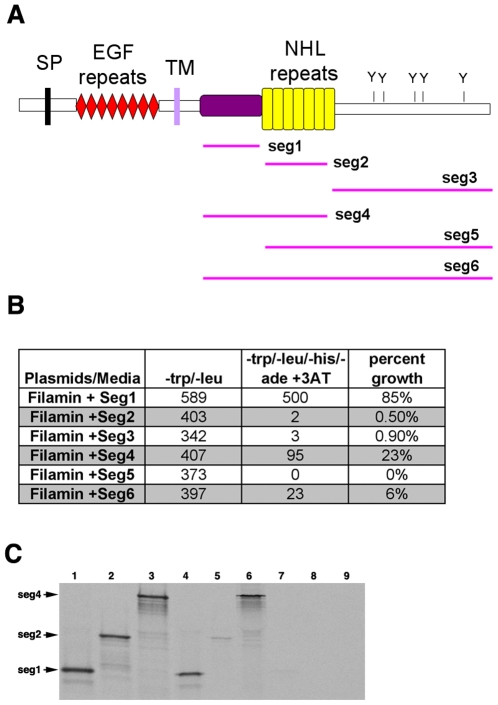
*Drosophila* Ten-m physically interacts with Filamin through its FID domain in yeast. (**A**) The domain structure of Ten-m. For deletion mapping of the filamin binding domain, the C-terminus was further truncated into six segments. SP: signal peptide. EGF: epidermal growth factor repeats. TM: transmembrane domain. NHL: domain shared by Ncl1, Hta2 and Lin41. Y: putative tyrosine phosphorylation site. (**B**) Deletion mapping of the filamin interaction domain of Ten-m by yeast co-transformation. The leftmost column shows the individual Ten-m segment being co-transformed with Filamin into the AH109 yeast. Seg1, showing the highest percentage, is sufficient for interacting with Filamin. (**C**) GST pull down assay. *In vitro* translated Ten-m seg1, seg2 and seg4 were labeled with ^35^S. The GST-Filamin fusion protein efficiently pulls down seg1 and seg4 (which includes seg1). Lanes 1–3: seg 1; 2 and 4 input. Lanes 4–6: GST-Filamin plus seg 1; 2 and 4. Lanes 7–9: GST plus seg 1; 2 and 4.

To narrow down the part of Ten-m that is sufficient for the interaction with Filamin, the Ten-m encoding region was divided into six segments (Figure 5A). The predicted NHL repeats, which were previously reported to be putative protein-protein interaction domains [25] are present in segment 2 (seg 2). The protein sequences upstream and downstream of seg 2 were designated seg 1 and seg 3, respectively. As shown in Figure 5B (and Supporting Information S1), seg1 showed the strongest interaction whereas seg4 showed weaker interactions, suggesting that seg1 may be sufficient for Ten-m to bind Filamin. To provide additional evidence for this interaction, we carried out a GST pull down assay using a GST-Filamin fusion protein, and ^35^S labeled Ten-m seg1, seg2 and seg4. Under these conditions, seg1 and seg4 bound the GST-Filamin fusion protein much more efficiently than seg2 (Figure 5C), indicating that seg1 is sufficient for the interaction. Thus, *Drosophila* Ten-m interacts with Filamin and a region in seg1 is mostly responsible for this interaction. Seg1 corresponds to the cysteine rich region between the EGF-like repeat domain and the NHL repeat domain, from amino acids 910 to 1173. Together with the EGF and NHL repeat domains, this previously undefined region is the most highly conserved between Ten-m/Odz family members. We named seg1 the Filamin interacting domain (FID).

### Filamin is necessary for proper peripheral motor axon guidance

**Figure 6:**
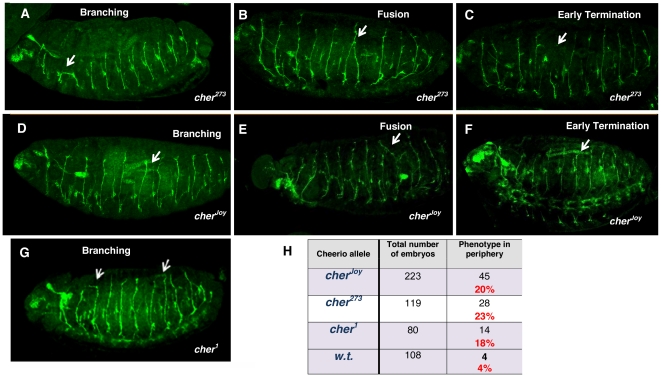
Cheerio mutants have embryonic motor neuron axon guidance defects. Fasciclin II expression in homozygous mutant embryos for *cheerio* (Filamin). (**A–C**) *cher^273^*; (**D–F**) *cher^joy^*; (**G**) *cher^1^*. The aberrant axon morphologies seen in peripheral axons are much like those seen for *Ten-m* mutant embryos. All embryos are: head, left; dorsal, top. (**H**) Percentage of embryos showing at least one aberrant axon phenotype. *cher^1^* and *cher^273^* are viable, female sterile alleles propagated with balancer chromosomes. *cher^joy^* is viable and fertile for which all offspring are homozygotes.

Filamin is encoded by the *cheerio* locus [41], for which several mutations have been isolated. We examined embryos homozygous for *cher^joy^*, and for the female sterile alleles *cher^1^*, and *cher^273^*. Embryos homozygous for these *cheerio* alleles have ISN projections that exit the CNS normally, but display peripheral neuron motor axon defects (Figure 6A–G) of the types observed for *Ten-m* mutant alleles. The aberrant patterns include ISNs that cross the segmental boundary and fasciculate with adjacent ISNs (Figure 6A–B, G), including those that defasciculate before crossing the segment boundary to fasciculate with an adjacent ISN (Figure 6D–E). Also included are ISNs that do not reach the terminal domain (Figure 6C, F). The frequency of defects is significant for all three alleles (Figure 6H). From crosses between homozygous *cher^joy^* females and *cher^273^* males, 18% of the resulting transallelic *cher^273^/cher^joy^* embryos bore axon guidance defects. The nature and frequency of the phenotypes were like those of the 20% observed for *cher^joy^* homozygous embryos. These data provide *in vivo* evidence that Filamin and Ten-m affect growth cone guidance of motor neurons.

### Epidermal Ten-m and Filamin expression overlap, and might influence motor axon guidance

**Figure 7:**
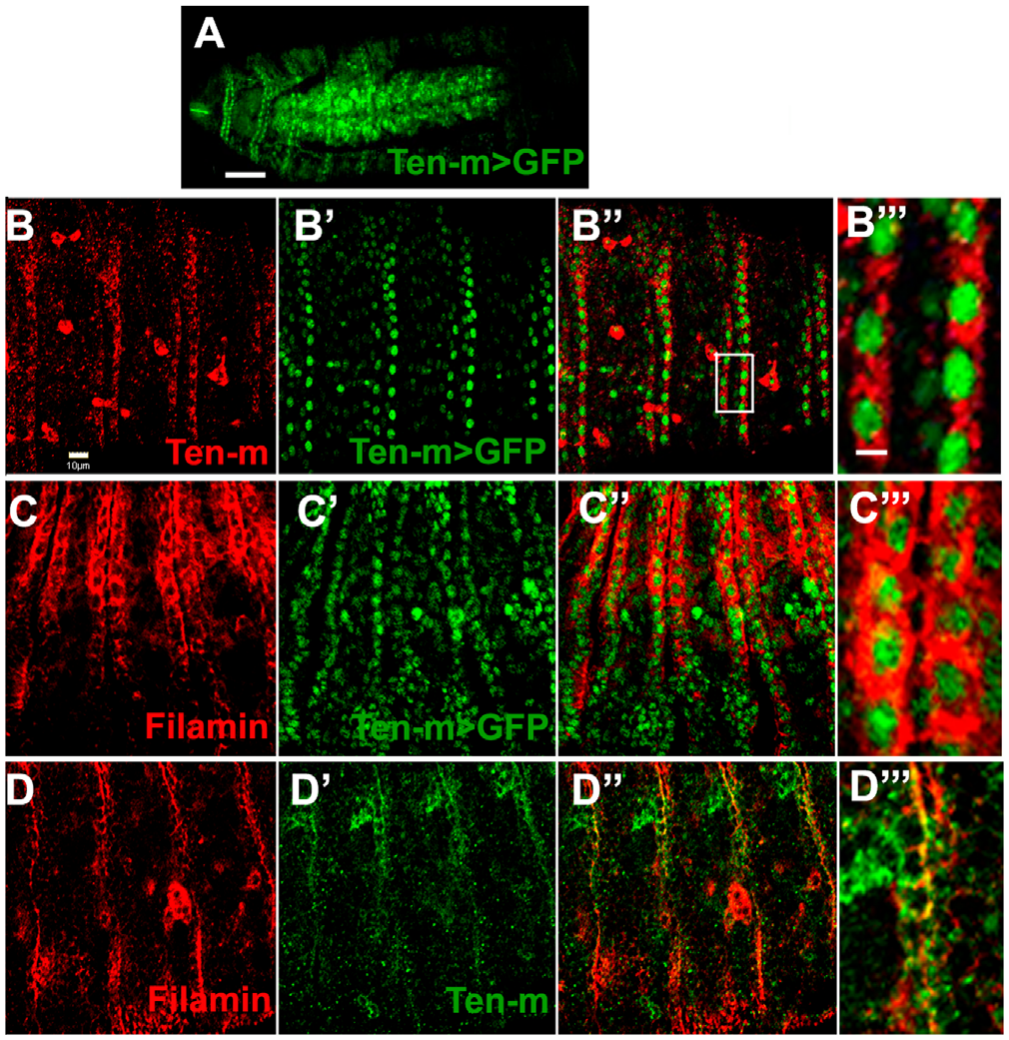
Filamin and Ten-m expression overlap in epidermal belts. (**A**) The *Ten-m^CB04362^* enhancer trap expresses *Ten-m* driven GFP in double rows of epidermal cell nuclei at segment boundaries, and in the CNS at stage 16. (**B–D**) Ten-m and Filamin are co-localized in the epidermal stripes in stage 15 and early stage 16 embryos. The embryos are shown as lateral views: anterior - left, dorsal - up. (**B-B′′′**) Ten-m protein (red) and *Ten-m^CB04362^* enhancer trap expression (green) appear in the same cells, in the cytoplasm and nucleus, respectively. Double rows of epidermal cells express Ten-m, as do a number of scattered hemocytes. (**B′′′**) is an enlargement of the area marked in the merged image of (**B′′**). (**C-C′′′**) Filamin (red) and *Ten-m^CB04362^* enhancer trap expression (green) appear in the same cells, in the cytoplasm and nucleus, respectively. Double rows of epidermal cells express the highest levels of Filamin. (**C′′′**) is an enlargement from (**C′′**). (**D-D′′′**) Filamin protein (red) and Ten-m protein (green) appear in the same epidermal cells in a cytoplasmic and cell surface distribution. (**D′′′**) is an enlargement from (**D′′**). Scale bars are: 40 µm in A, 10 µm in B, and 2 µm in B′′′.

Expression patterns for Ten-m and for Filamin suggest that the two interacting proteins might be expressed in the same ectodermal cells in restricted patterns during embryonic development. At stage 8, Ten-m and Filamin are expressed in all furrows and the posterior midgut, although Filamin is expressed more widely than Ten-m. Like Ten-m, Filamin is expressed in a repetitive pattern, at stage11. By stage 13, Ten-m is abundant in the ventral nerve cord while the repetitive expression pattern of Filamin mostly disappears, to then be expressed in the epidermis. At stage 15, Ten-m is expressed in the axons of the CNS as well as in epidermal stripes. Filamin is also expressed in epidermal stripes at stage 15. At high resolution, the overlap of the stripes includes distinct domains of cellular co-expression of these two proteins (Figure 7).

Double staining of the two proteins establishes the cell-by-cell stripewise epidermal co-expression of these proteins (Figure 7D). A high resolution assessment of the proteins' co-expression is best demonstrated with a nuclear GFP exon trap of an insertion into the 5-prime UTR of *Ten-m*, *Ten-m^CB04632^* (Figure 7A) [35]. In *Ten-m^CB04632^* embryos, cytoplasmic Ten-m protein imaged by immunofluorescence, and endogenous nuclear GFP *Ten-m* reporter expression occur in the same cells (Figure 7B). This indicates that the nuclear GFP faithfully represents the expression of Ten-m. When anti-Filamin immunofluorescence of these embryos is imaged, the cytoplasmic *cheerio* encoded protein product co-localizes to cells expressing the nuclear GFP in distinct rows of epidermal cells (Figure 7C). This co-localization data indicate that during *Drosophila* embryonic development, Ten-m and Filamin are co-expressed in belts of epidermal cells at segmental boundaries at embryonic stage 15. ISN motor axon projections migrate out of the CNS at embryonic stage 14 and reach the dorsal muscle terminus at stage 16 [10]. Ten-m and Filamin epidermal expression, including co-expression of Ten-m and Filamin in epidermal stripes (Figure 7C, D) at stage 15, may impact motor axon navigation. These data, in light of the Ten-m ectopic expression data, suggest that spatially restricted expression of these genes in epidermal cells may play a role in helping to ensure accurate axonal guidance.

## Discussion

In this study, we characterized new alleles of *Ten-m* that were generated in a large scale P element mutagenesis project [32] to find that mutant *Ten-m* does not cause segmentation defects, but leads to abnormal motor axon navigation. We also identified Filamin as an interacting partner of Ten-m, and showed that mutations in the gene encoding Filamin lead to the same motor axon phenotypes those of *Ten-m*. The data suggest that Filamin and Ten-m may function together to influence motor axon migration.

### *Ten-m* in body pattern formation

*Ten-m* was thought to be the first non-transcription factor pair-rule gene [19], [21]. Three P-element insertion alleles were reported to lie in the same complementation group and be embryonic lethal. One allele, *5309*, showed a severe pair-rule phenotype, one allele showed a moderate version of the same phenotype, whereas other lethal alleles did not have a significant cuticle phenotype [19], [21]. Here we show that the cuticle phenotype associated with the original *5309* stock segregates with the balancer chromosome. Indeed, the *Df(3L)Ten-m^AL1^* and *Df(3L)Ten-m^AL29^* deletion alleles exhibit fusions similar to the *5309* allele when balanced with the same balancer chromosome as the one used in 5309 (Figure 2). Our data show that the “*Ten-m*” cuticle phenotype analyzed previously is an *odd-paired* mutation on the balancer chromosome and is not due to the *Ten-m* mutation itself. It should be noted that the embryonic CNS longitudinal connective discontinuity phenotype documented [19] for *Ten-m^5309^* is also due to a mutation on that original balancer.

### *Ten-m* in motor axon migration

When Ten-m is not expressed, or is ectopically expressed in the ectoderm, aberrant motor axon growth cone guidance occurs. Growth cones use various kinds of substrates and guidance cues to navigate through a specific path to find their targets [4]. In *Drosophila* and *C. elegans*, genetic screens have identified many secreted or transmembrane guidance cues including Netrins, Semaphorins, Slits, Nephrins, and classic morphogens that also act as guidance molecules [10], [53], [54], [55]. Most of these cues are expressed either by axonal tracts themselves or along the axonal trajectory by peripheral tissues, such as muscles. We have identified a transmembrane protein affecting migration, Ten-m, which is expressed in epidermal stripes and in neurons. In both vertebrate and invertebrate embryos, axons must first exit the CNS, and then migrate along stereotyped pathways to reach their specific targets in the periphery. Since motor axons in both *Ten-m* loss of function mutants, and Ten-m ectopic expression embryos, exit the CNS normally but do not migrate along their specific paths (Figure 3 and Figure 4), Ten-m appears to be dispensable for axonal extension but necessary for pathfinding decisions in the periphery.

Ten-m is often required for correct choice point determination. During embryonic development, axons preferably extend along the surface of other axons to form axon bundles or fascicles (selective fasciculation), and exit those fascicles to navigate into their targets (selective defasciculation). These processes are regulated by both attractive and repulsive cues [49]. These attractive and repulsive molecules can originate from the axonal tracts themselves or from the surrounding peripheral tissues [6]. A guidance cue disruption of a repulsive molecule should cause abnormal defasciculation. In the case of Ten-m loss of function mutations, or gain of function, the ISN branches fail to maintain their segmental boundaries and invade the adjoining segments, occasionally to fuse with the adjacent segmental nerve (Figure 3 C, D and E). In mutants, loss of Ten-m activity in the motor axons could lead to failing to respond properly to cues, among other possible failings of the neurons. However, epidermal ectopic, or Paired-driven Ten-m expression leading to the same phenotypes suggest that Ten-m impacts peripheral cues, assuming that the overexpression effects were specific.

Axon guidance disruptions observed when Ten-m is ectopically expressed suggests that Ten-m either: maintains peripheral cells to allow them to reach a stage to express a cue for motor axons; or more directly regulates the expression of cues to which motor axons respond. We speculate that Ten-m might itself act as a peripheral cue for migration. We suggest that epidermal Ten-m, and perhaps specifically its expression spatially restricted to stripes, could help position a repulsive guidance cue for the ISN axons and prevent them from crossing into the adjacent segments. Given the collection of defects observed in motor neuron axons, Ten-m might induce a gene product that is, or might itself be, both a repulsive and an attractive guidance molecule, a situation that is not uncommon. For example, Netrins were first found as a chemoattractant for vertebrate commissural axons [56] and circumferential axons in *C. elegans* [54], [57]. However, Netrins can also repel some axons, as demonstrated in unc-6/netrin mutant *C. elegans* and *Drosophila* [54], [58]. *DCC/frazzled* (deleted in colorectal carcinoma), a netrin receptor, mediates both attraction and repulsion [59] while UNC-5, another netrin receptor, functions exclusively in repulsion [59], [60], [61].

### Filamin and axon guidance

Filamins are very large proteins with an actin binding domain and more than 20 Ig-like repeats, that self associate as dimers. They act as Actin crosslinking proteins that are also scaffolds for a very large number and variety of binding partners. As such, they are involved in many functions, but especially relevant are cell adhesion and migration. This includes interactions with different cytoskeletal complexes. In flies, Filamin affects peripheral motor axon navigation in a manner similar to that of Ten-m (Figure 6). This function echoes vertebrate filamin activities. In contrast to *Drosophila*, mammals have three filamin proteins, A, B and C. Loss of function mutations in Filamin A are found in the human disease periventricular heterotopia, which is a defect in axonal migration that has been associated with the dynamic regulation of actin [62], [63], [64], [65]. However, detailed studies of patients carrying mutations shows evidence for a more complex regulation of axonal navigation than can be explained by simply an effect on growth cone motility [66], [67], [68]. Our studies in flies suggest that in addition to growth cone motility, the context and restricted expression pattern of Filamin might influence axon guidance. In *Drosophila*, Filamin associates with the seg1 domain of Ten-m, a highly conserved domain within the Ten-m/Odz family that we have named the FID (Figure 5). These two proteins are expressed in the epidermis, including co-expression in a series of ‘belts’ of epidermal cells, strongest laterally (Figure 7).

How might Ten-m, together with Filamin, regulate axonal guidance? We hypothesize that anchored epidermal Ten-m and Filamin might influence lateral motor axon navigation. These two proteins might set the stage for proper development leading to the expression of spatially restricted epidermal axon guidance cues, or directly impact the regulation of such cues, as ISN motor axon projections start to migrate out of the CNS and begin to reach their lateral and dorsal muscle targets. One speculation is that Ten-m linked to Filamin could itself be a candidate cue for motor axons.

## Supporting Information

Supporting Information S1 Supporting figures.
(DOC)
