# Supplementary material for: Drosophila Ten-m and Filamin Affect Motor Neuron Growth Cone Guidance
Source: PLoS One. 2011 Aug 8;6(8):e22956. doi: 10.1371/journal.pone.0022956 (PMC3152545; doi:10.1371/journal.pone.0022956)
Supplement: Supporting Information S1 — Supporting figures. (DOC) [file pone.0022956.s001.doc]

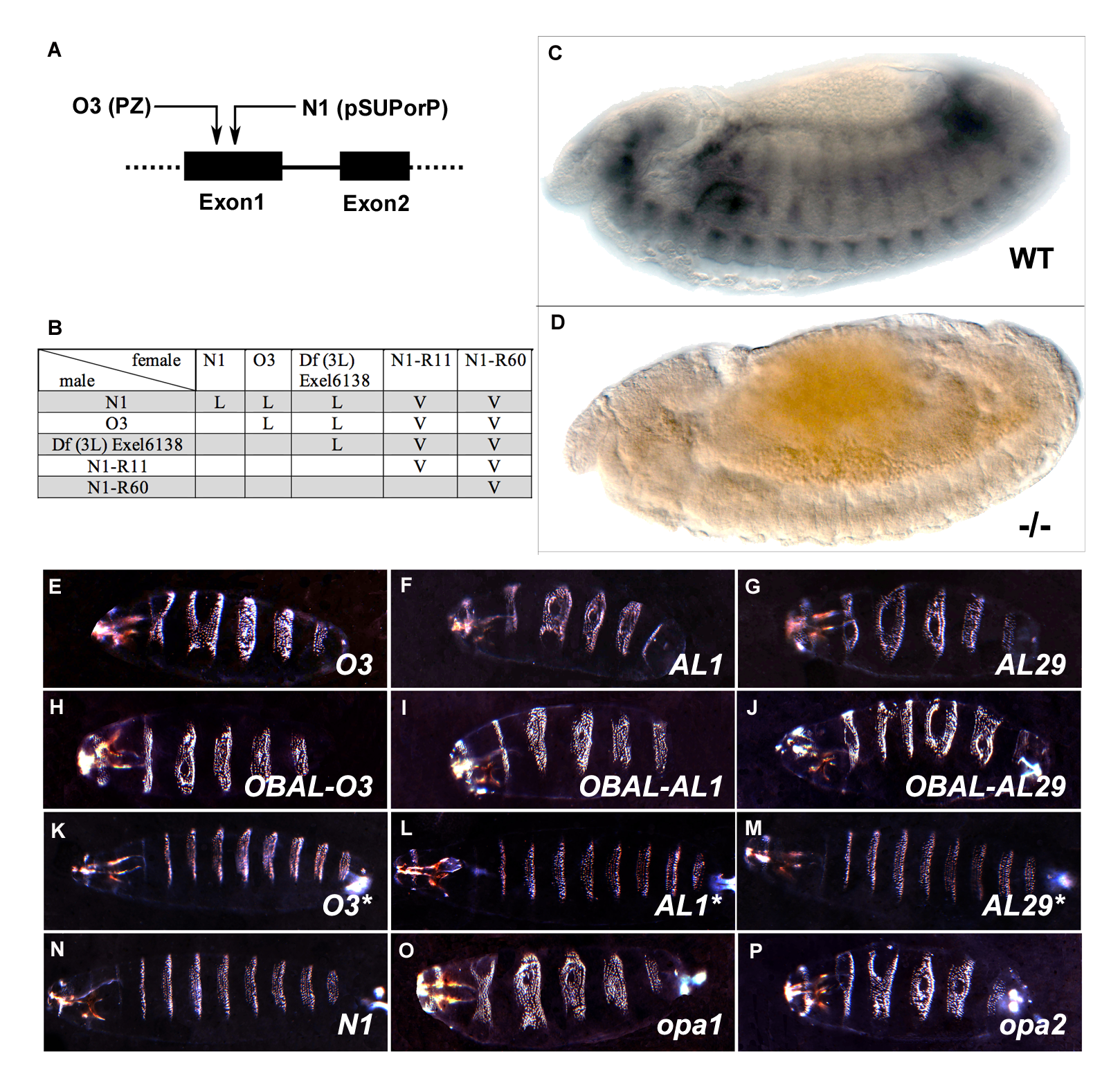


**A**

**B**

**Supporting Information Figure 1. *Ten-m* allele *N1* produces no detectable Ten-m protein.**

Ten-m expression was assessed by embryonic immunostaining with monoclonal antibody Mab113, raised against aa 56-251 of the Ten-m protein (Baumgartner et al., 1993). Ten-m is expressed in epidermal stripes and the central nervous system at stage 15 in wild type embryos (A). Ten-m protein is not expressed in *N1* mutant embryos (B). *N1* immunostaining is unlike wild type embryos, but similar to the *5309* allele, which displays no detectable protein (Levine et al., 1994; Dgany and Wides, 2002). Hence, both P-element insertions, located within exon 1, disrupt Ten-m protein production.


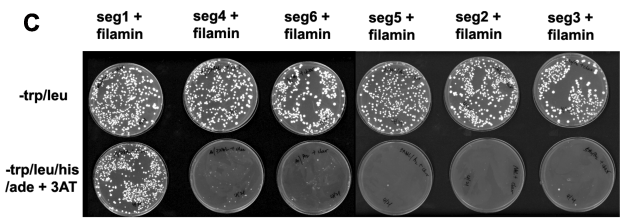


**Supporting Information Figure 2. Filamin identified as an interacting partner of Ten-m.** **A**. Generation of the bait protein. The C-terminus of ten-m was cloned into pGBKT7 vector and the predicted 200 kD Gal4 DNA binding domain fusion was expressed in yeast as detected by anti-Myc antibody by Western blot. The control is protein extract from non-transformed yeast. **B**. Filamin interacts with ten-m. Shown here are different combinations of transformed yeast streaked on quadruple dropout media. The yeast co-transformed with filamin and ten-m grows very well on the quadruple dropout media. Area #1: empty pGBKT7 vector transformed yeast strain AH109. Area #2: filamin-pACTII transformed AH109. Area #3: filamin-pACTII and ten-m-pGBKT7 co-transformed AH109. Area #4: ten-m-GBKT7 transformed AH109. **C.** Deletion mapping of Ten-m by co-transformations cultured on different media. The same amount of co-transformed cells were plated on –trp/leu and –trp/leu/his/ade +3AT media (see Materials and methods).
